# Efficacy of Lianhua Qingwen Compared with Conventional Drugs in the Treatment of Common Pneumonia and COVID-19 Pneumonia: A Meta-Analysis

**DOI:** 10.1155/2020/5157089

**Published:** 2020-09-17

**Authors:** Caiyun Hu, Mingming Liang, Fengfeng Gong, Bin He, Dongdong Zhao, Guoliang Zhang

**Affiliations:** ^1^Department of Scientific Research, The First Affiliated Hospital of Anhui University of Traditional Chinese Medicine, 117 Meishan Road, Hefei, Anhui, China; ^2^Epidemiology and Health Statistics, School of Public Health, Anhui Medical University, No. 81 Meishan Road, Hefei, Anhui, China; ^3^Fuyang Hospital of Anhui Medical University, No. 99 Huangshan Road, Fuyang, Anhui, China; ^4^Infection Department, The First Affiliated Hospital of Anhui Medical University, No. 218 Jixi Road, Hefei, Anhui, China

## Abstract

**Methods:**

Seven English and Chinese databases were used to search for qualified experimental studies as of July 27, 2020. All data were extracted directly from the included studies, and no special conversion formula was used. The weighted mean difference (WMD), 95% confidence interval (CI), and odds ratio (OR) were used for evaluation.

**Results:**

Forty-two studies involving 3793 subjects met the qualification criteria. For common pneumonia, a short duration of flu-like symptoms (WMD = −1.81, 95% CI = −2.12 to −1.50, *P* < 0.001), sputum (WMD = −1.10, 95% CI = −1.50 to −0.70, *P* < 0.001), pulmonary rale (WMD = −2.03, 95% CI = −2.74 to −1.31, *P* < 0.001), pulmonary imaging improvement (WMD = −1.88, 95% CI = −2.28 to −1.47, *P* < 0.001), curative effect (OR = 3.65, 95% CI = 2.81 to 4.76, *P* < 0.001), and healing period (WMD = −1.68, 95% CI = −2.62 to −0.74, *P* < 0.001) were associated with the Lianhua Qingwen group; subgroup analysis based on flu-like symptoms showed statistically significant improvements in fever and cough. For COVID-19 pneumonia, improvements in flu-like symptoms (OR = 3.18, 95% CI = 2.36 to 4.29, *P* < 0.001), shortness of breath (OR = 10.62, 95% CI = 3.71 to 30.40, *P* < 0.001), curative effect (OR = 2.49, 95% CI = 1.76 to 3.53, *P* < 0.001), healing period (WMD = −2.06, 95% CI = −3.36 to −0.75, *P* = 0.002), and conversion of severe cases (OR = 0.46, 95% CI = 0.27 to 0.77, *P* = 0.003) were associated with the Lianhua Qingwen group; subgroup analysis indicated statistically significant improvements of fever, cough, fatigue, and muscle pain in the Lianhua Qingwen group compared to the conventional drug group. Regarding adverse reactions, no significant difference was detected for common pneumonia (OR = 0.75, 95% CI = 0.54 to 1.05, *P* = 0.097).

**Conclusions:**

Lianhua Qingwen combined with conventional drugs may be a promising therapy for treating common pneumonia and COVID-19 pneumonia.

## 1. Introduction

Traditional Chinese medicine, with a thousand years of history in China, has been used to treat various diseases [1–5]. Lianhua Qingwen (连花清瘟), which is obtained by the reduction and addition of combined Maxing Shigan decoction and Yin Qiao powder, has been widely accepted as a broad-spectrum antiviral agent in the clinic [6]. It consists of 13 herbs: Lian Qiao (Fructus Forsythiae, weeping forsythia capsule, 连翘), 255 g; Ma Huang (*Herba Ephedrae*, ephedra, 麻黄), 85 g; Jin Yin Hua (*Flos Lonicerae*, honeysuckle flower, 金银花), 255 g; Ban Lan Gen (*Radix Isatidis*, isatis root, 板蓝根), 255 g; Mianma Guanzhong *(Rhizoma Dryopteris crassirhizomae*, male fern rhizome, 绵马贯众), 255 g; Bo He (*Herba Menthae*, menthol, 薄荷), 7.5 g; Shi Gao (*Gypsum Fibrosum*, gypsum, 石膏), 255 g; Guang Huo Xiang (*Herba Pogostemonis*, cablin patchouli herb, 广藿香), 85 g; Hong Jing Tian (*Herba Rhodiolae*, rose-boot, 红景天), 85 g; Yu Xing Cao (*Herba Houttuyniae*, heartleaf houttuynia herb, 鱼腥草), 255 g; Da Huang (*Radix et Rhizoma Rhei,* rhubarb root and rhizome, 大黄), 51 g; Ku Xing Ren (*Semen Armeniacae Amarum*, bitter apricot seed, 苦杏仁) 85 g; and Gan Cao (*Radix Glycyrrhizae*, liquorice root, 甘草), 85 g [7]. Lianhua Qingwen, which was approved by China Food and Drug Administration (CFDA) in 2004, was the first traditional Chinese medicine that entered the rapid drug approval channel of the CFDA and was used to prevent severe acute respiratory syndrome (SARS). It has been widely used for more than ten years and has become a representative traditional Chinese medicine for respiratory infectious diseases. Pneumonia is one of the most common infections in individuals of all ages, and morbidity of pneumonia increases with age [8]; it is also a significant cause of global mortality. In December 2019, a group of patients with typical pneumonia symptoms of unknown pathogenic factors was described in China, and patients with similar symptoms were quickly detected in other regions. The official name was announced as coronavirus disease 2019 (COVID-19) by the World Health Organization (WHO) on February 11, 2020. This virus has been a major emerging global public health event with no antiviral treatment or vaccine [9]. As of August 6, 2020, the Situation Report-199 of the WHO reported 18,614,177 cases of confirmed COVID-19 pneumonia and 702,642 deaths worldwide [10]. In addition to its impact on health, the COVID-19 pandemic has also led to social, economic, and political damage [11]. The National Health Commission of the People's Republic of China published guidelines for the diagnosis and treatment of COVID-19 pneumonia (Trial version from the Fourth/Fifth/Sixth/Seventh Edition) and recommended Lianhua Qingwen as a traditional Chinese medicine for COVID-19 pneumonia [12]. Some studies have suggested that Lianhua Qingwen has a therapeutic effect on pneumonia, including COVID-19 pneumonia. However, no consensus has been reached. Therefore, we performed a meta-analysis to investigate and compare the therapeutic effect of Lianhua Qingwen with conventional drugs on pneumonia symptoms.

## 2. Materials and Methods

### 2.1. Selection Criteria

An electronic search of PubMed, Embase, the Cochrane Library, Foreign Medical Literature Retrieval Service (FRMS), Wanfang (a Chinese database), VIP (a Chinese database), and CNKI (a Chinese database) was performed from the date of inception until July 27, 2020. For the English databases, the following terms were searched in the title, keywords, or abstract: (“Lianhuaqinwen” or “Lianhua Qingwen”) and (“Pneumonia” or “Lung Inflammation” or “Pulmonary Inflammation” or “COVID-19” or “Coronavirus disease” or “Novel coronavirus”). For the Chinese databases, the search terms were “Lianhuaqingwen” and (“Feiyan” or “Feibuyanzheng” or “COVID-19” or “Guanzhuangbindu”).

### 2.2. Study Inclusion and Exclusion Criteria

Studies were selected based on the following criteria: (1) experimental studies; (2) pneumonia was diagnosed according to clinical symptoms, laboratory tests, chest X-ray results, or relevant diagnostic criteria; (3) the Lianhua Qingwen group was treated with Lianhua Qingwen or Lianhua Qingwen combined with conventional drugs (the conventional drug group was given conventional antibiotics, antiviral drugs, or symptomatic treatment); and (4) relevant data of the efficacy index (i.e., flu-like symptoms (fever, cough, sore throat, nausea, diarrhoea, loss of appetite, fatigue, muscle pain, and headache); sputum; pulmonary rales; shortness of breath; breathlessness; chest tightness; pulmonary imaging improvement; curative effect; healing period; conversion of severe cases; and adverse reactions) were provided. The following types of studies were excluded: letters, case reports, those with duplicate or incomplete data (including those in which the efficacy index did not meet the inclusion criteria), reviews or commentaries, animal-based studies, irrelevant studies, and those with a non-Western medicine control.

### 2.3. Data Extraction and Quality Evaluation

The following information was collected: the first author's name, year of publication, age, sex, sample size, efficacy index, and adverse reactions. All data were extracted directly from the included studies, and no special conversion formula was used. Continuous variables included the total number of patients with symptoms (reported as the mean and standard deviation), and categorical variables included the number of patients with symptoms and the number of patients without symptoms. Disagreements were resolved by a panel discussion with other reviewers. The quality of the eligible studies was evaluated by the CONSORT statement [13] and the Jadad scale [14], which considered sample size determination, randomization, blinding, and loss to follow-up/withdrawal; scores of 0–2, 3–4, and 5–7 were rated as low, moderate, and high quality, respectively. The Cochrane Collaboration risk of bias tool [15], which consists of seven items, was used to classify each item as low risk of bias, high risk of bias, and unclear risk of bias.

### 2.4. Statistical Analysis

Statistical analysis was performed using Stata 14 (Stata Corporation, College Station, TX, USA). Continuous variables are expressed as the weighted mean difference (WMD) and 95% confidence interval (CI), and categorical variables are expressed as the odds ratio (OR) and 95% CI. Heterogeneity was evaluated with the *I*^2^ statistic: when *I*^2^ > 50%, indicating obvious heterogeneity, a random-effects model was used; otherwise, a fixed-effects model was used [16, 17]. Subgroup, sensitivity, and regression analyses were used to explore possible sources of heterogeneity. Subgroup analysis was performed based on the pneumonia type or flu-like symptoms. Sensitivity analysis was performed to assess the influence of each study on a pooled estimate by excluding studies individually and rerunning the meta-analysis. Publication bias was assessed by Begg's test and Egger's test, and visual estimation (via a funnel plot) was also considered; if bias existed, the “trim and fill” method was used to assess publication bias [18]. A *P* value <0.05 was considered statistically significant.

## 3. Results

### 3.1. Search Results

**Figure 1:**
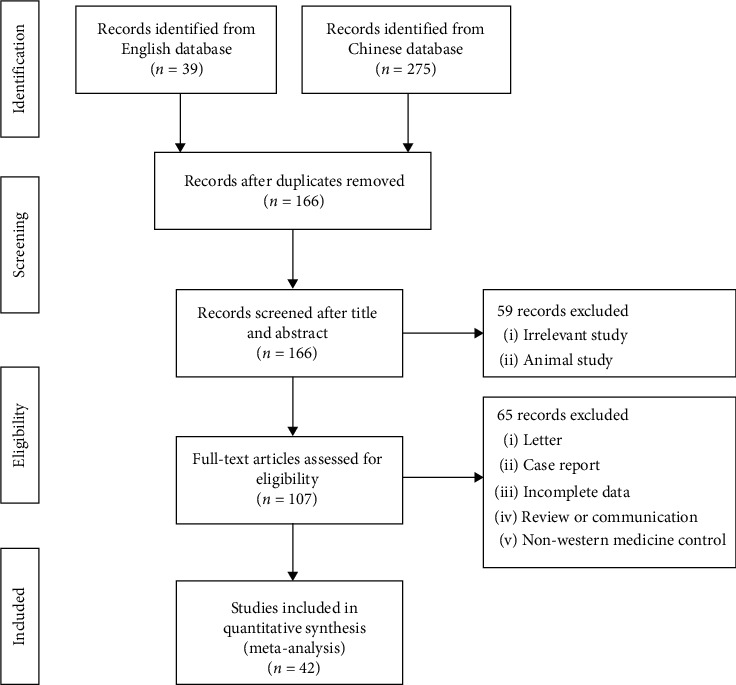
Flowchart of the search and selection process.

**Figure 2:**
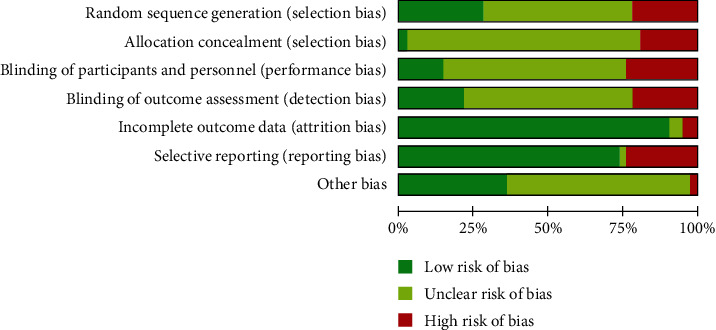
Percentage of risk of bias for all qualified studies.

The detailed flowchart of article selection is presented in Figure 1. A total of 314 citations were identified, and of these, 148 duplicate citations were removed; 59 citations were excluded for irrelevant or animal studies by screening the title and abstract. After full-text screening of the remaining 107 citations, an additional 65 citations were excluded due to the reasons given in Figure 1. Ultimately, 42 experimental Chinese studies involving 3793 subjects were subjected to the meta-analysis. The publication year ranged from 2012 to 2020 [19–60]. Of these studies, 35 researched common pneumonia (including viral pneumonia, mycoplasma pneumonia, community-acquired pneumonia, bronchial pneumonia, bacterial pneumonia, or pneumonia), and the remaining 7 studies researched COVID-19 pneumonia (diagnosed according to the National Health Commission of the People's Republic of China published guidelines for the diagnosis and treatment of COVID-19 pneumonia: Trial version from the Fourth/Fifth Edition). The control groups were given conventional antibiotics, antiviral drugs, or symptomatic treatment. The main characteristics and quality scores of the eligible studies on common pneumonia and COVID-19 pneumonia are shown in Table 1. Regarding the risk of bias assessment, 33 studies reported randomization (of which 12 studies clearly described the randomized methods), and the remaining 9 studies were grouped according to interventions or the hospitalization order. Only one study reported allocation concealment and blinding method, since the symptom indicators needed to be measured by instruments or humans. Therefore, most studies with blinded participants and personnel and a blinded outcome assessment were classified as “unclear risk of bias.” Concerning incomplete outcome data and selective reporting, since most studies reported complete data on all symptom indicators, there was a low risk of attrition bias and reporting bias. The percentage of risk of bias is shown in Figure 2.

### 3.2. Efficacy Evaluation of Common Pneumonia

#### 3.2.1. Flu-Like Symptoms

**Figure 3:**
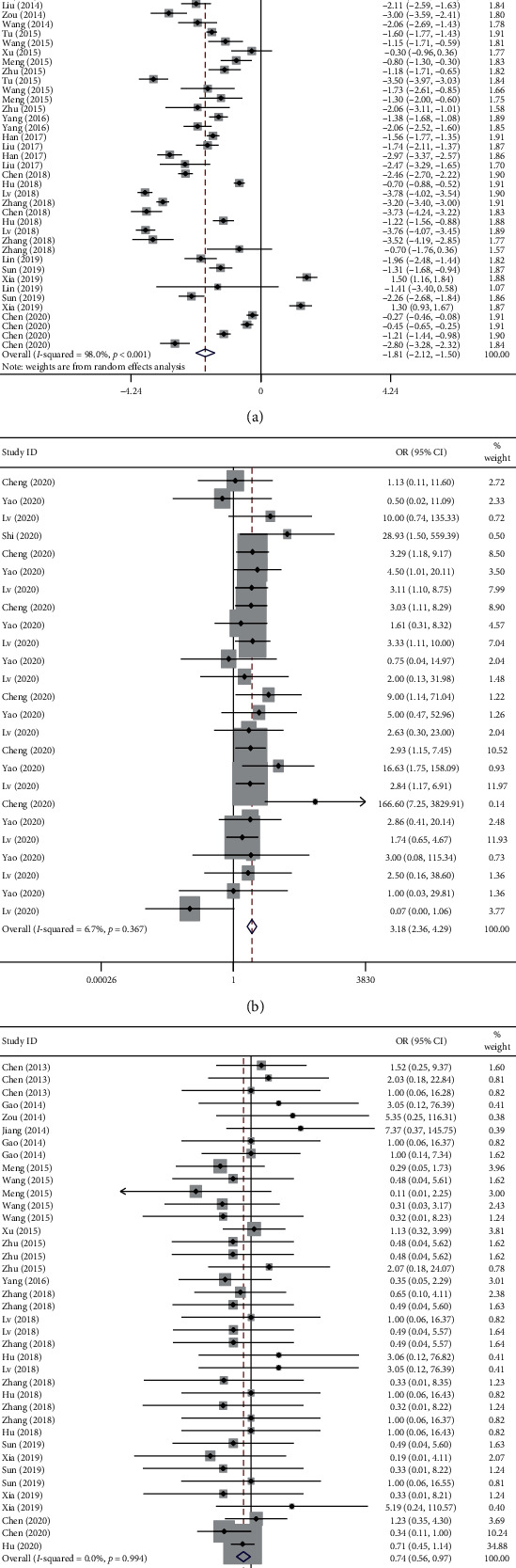
Forest plot of the comparison between the Lianhua Qingwen group and the conventional drug group on flu-like symptoms and adverse reactions: (a) flu-like symptoms for common pneumonia; (b) flu-like symptoms for COVID-19 pneumonia; (c) adverse reactions.

Thirty-one studies compared the duration of flu-like symptoms between the Lianhua Qingwen and conventional drug groups. Among these studies, 29 reported the symptom remission time for fever, and 26 reported the symptom remission time for cough. A short duration of flu-like symptoms was observed in the Lianhua Qingwen group (WMD = −1.81, 95% CI = −2.12 to −1.50, *P* < 0.001). Subgroup analysis showed significant differences in the durations of fever (WMD = −1.58, 95% CI = −1.99 to −1.17, *P* < 0.001) and cough (WMD = −2.07, 95% CI = −2.57 to −1.57, *P* < 0.001) between the Lianhua Qingwen and conventional drug groups.  Figure 3 shows a forest plot of flu-like symptoms for common pneumonia.

#### 3.2.2. Other Symptoms

 
*Sputum*. Four studies reported the effects of Lianhua Qingwen and those of conventional drugs on the duration of sputum. The duration of sputum was shorter in the Lianhua Qingwen group than in the conventional drug group (WMD = −1.10, 95% CI = −1.50 to −0.70, *P* < 0.001).
 
*Pulmonary Rale*. Eighteen studies reported an effect on the duration of pulmonary rale. The duration of fever was shorter in the Lianhua Qingwen group than in the conventional drug group (WMD = −2.03, 95% CI = −2.74 to −1.31, *P* < 0.001).
 
*Pulmonary Imaging Improvement*. Seven studies reported the duration of pulmonary imaging improvement. The difference between the Lianhua Qingwen group and the conventional drug group was statistically significant (WMD = −1.88, 95% CI = −2.28 to −1.47, *P* < 0.001).
 
*Curative Effect*. Thirty studies reported the curative effect of Lianhua Qingwen and that of conventional drugs. The difference between the Lianhua Qingwen group and the conventional drug group was statistically significant (OR = 3.65, 95% CI = 2.81 to 4.76, *P* < 0.001).
 
*Healing Period*. Twelve studies reported the healing period of the Lianhua Qingwen and conventional drug groups. The Lianhua Qingwen group had a shorter healing period than the conventional drug group (WMD = −1.68, 95% CI = −2.62 to −0.74, *P* < 0.001).

Results from the quantitative analysis of common pneumonia are shown in Table 2. Forest plots of other symptoms related to common pneumonia are shown in Figure S1.

### 3.3. Efficacy Evaluation of COVID-19 Pneumonia

#### 3.3.1. Flu-Like Symptoms

Four studies compared improving flu-like symptoms (fever, cough, sore throat, nausea, diarrhoea, loss of appetite, fatigue, muscle pain, and headache) between the Lianhua Qingwen and conventional drug groups. The difference in improving flu-like symptoms between the Lianhua Qingwen and conventional drug groups was statistically significant (OR = 3.18, 95% CI = 2.36 to 4.29, *P* < 0.001). Subgroup analysis indicated significant associations with fever (OR = 4.05, 95% CI = 1.78 to 6.59, *P* < 0.001), cough (OR = 3.43, 95% CI = 1.87 to 6.29, *P* < 0.001), fatigue (OR = 2.82, 95% CI = 1.44 to 5.53, *P* = 0.003), and muscle pain (OR = 5.01, 95% CI = 1.44 to 17.40 *P* = 0.011). However, subgroup analysis revealed nonsignificant associations with sore throat (OR = 0.31, 95% CI = 0.03 to 2.99, *P* = 0.314), nausea (OR = 1.98, 95% CI = 0.49 to 7.95, *P* = 0.336), diarrhoea (OR = 1.28, 95% CI = 0.18 to 9.30, *P* = 0.810), loss of appetite (OR = 6.05, 95% CI = 0.69 to 53.14, *P* = 0.104), and headache (OR = 2.67, 95% CI = 0.30 to 23.83, *P* = 0.378).  Figure 3 shows a forest plot of flu-like symptoms for COVID-19 pneumonia.

#### 3.3.2. Other Symptoms

 
*Sputum*. Three studies reported the improvement in sputum. The difference in sputum improvement was statistically significant between the Lianhua Qingwen and conventional drug groups (OR = 4.30, 95% CI = 1.01 to 18.22, *P* = 0.048).
 
*Shortness of Breath*. Three studies compared the effect of Lianhua Qingwen with that of conventional drugs on the shortness of breath, and Lianhua Qingwen was associated with a better effect than conventional drugs (OR = 10.62, 95% CI = 3.71 to 30.40, *P* < 0.001).
 
*Breathlessness*. Three studies reported improvement in breathlessness in the Lianhua Qingwen and conventional drug groups. However, it failed to identify a statistically significant difference between the two groups (OR = 4.81, 95% CI = 0.97 to 23.86, *P* = 0.055).
 
*Chest Tightness*. Three studies reported the curative effect of chest tightness. A statistically significant difference was identified between the Lianhua Qingwen group and the conventional drug group (OR = 3.02, 95% CI = 1.23 to 7.42, *P* = 0.016).
 
*Pulmonary Imaging Improvement*. Five studies reported improvement in pulmonary imaging. A statistically significant difference was identified between the Lianhua Qingwen group and the conventional drug group (OR = 1.77, 95% CI = 1.29 to 2.42, *P* < 0.001).
 
*Curative Effect*. Five studies compared the curative effect of Lianhua Qingwen with that of conventional drugs. A statistically significant difference was identified between the two groups (OR = 2.49, 95% CI = 1.76 to 3.53, *P* < 0.001).
 
*Healing Period*. Two studies reported the healing period of the Lianhua Qingwen and conventional drug groups. The Lianhua Qingwen group was associated with a shorter healing period than the conventional drug group (WMD = −2.06, 95% CI = −3.36 to −0.75, *P* = 0.002).
 
*Conversion of Severe Cases*. Four studies reported the conversion of severe cases in both groups. A statistically significant difference was detected between the two groups (OR = 0.46, 95% CI = 0.27 to 0.77, *P* = 0.003).

Results from the quantitative analysis of COVID-19 pneumonia are shown in Table 2. Forest plots of other symptoms for COVID-19 pneumonia are shown in Figure S2.

### 3.4. Adverse Reactions

Eighteen studies (17 for common pneumonia and one for COVID-10 pneumonia) reported adverse reactions, including diarrhoea, rash, gastrointestinal reaction, headache, nausea, vomiting, abnormal liver function, and renal insufficiency, of the Lianhua Qingwen and conventional drugs groups. A significant statistical difference was detected between the two groups (OR = 0.74, 95% CI = 0.56 to 0.97, *P* = 0.029). Subgroup analysis revealed no significant difference regarding common pneumonia (OR = 0.75, 95% CI = 0.54 to 1.05, *P* = 0.097). Since only one study of COVID-19 pneumonia reported adverse reactions, no subgroup analysis was performed. Results from the quantitative analysis of adverse reactions are shown in Table 2.  Figure 3 shows a forest plot of adverse reactions.

### 3.5. Sensitivity Analysis and Meta-Regression

Sensitivity analysis was performed by deleting individual studies to assess the stability of the results. For common pneumonia, there was no significant alteration in any efficacy evaluation index. For COVID-19 pneumonia, changes in the following pooled results were observed: improvement in sputum (WMD = 4.30, 95% CI = 0.36 to 47.40), chest tightness (OR = 3.02, 95% CI = 0.77 to 28.11), and pulmonary imaging improvement (OR = 1.77, 95% CI = 0.95 to 3.41). Sensitivity analysis revealed changes in adverse reactions (OR = 0.74, 95% CI = 0.54 to 1.05). The sensitivity analysis results are shown in Table 3.

For common pneumonia, high heterogeneity was found in the following indexes: flu-like symptoms (*I*^2^ = 98.0), sputum (*I*^2^ = 62.2), pulmonary rales (*I*^2^ = 97.1), pulmonary imaging improvement (*I*^2^ = 56.5), and healing period (*I*^2^ = 97.0). However, subgroup analysis did not indicate heterogeneity, and no source of heterogeneity was found in the sensitivity analysis. Therefore, a meta-regression was performed to explore the likely source of heterogeneity, such as publication year, sample size, and quality score. The results indicated that these covariates could not explain all the heterogeneity and that the heterogeneity might be related to the inconsistency of conventional drugs used in each study and the individual absorption of drugs. For common pneumonia, high heterogeneity was found in the sputum index. Since there are limited studies on COVID-19 pneumonia, meta-regression was not performed. The results of the meta-regression are summarized in Table 4.

### 3.6. Publication Bias

**Figure 4:**
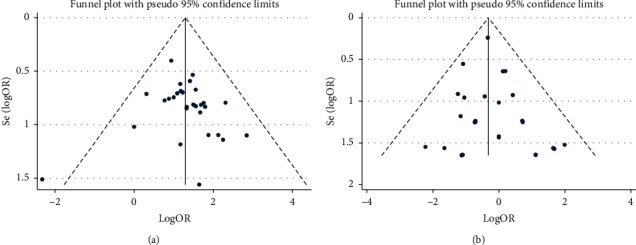
Funnel plots used to assess publication bias: (a) the index of curative effect for common pneumonia; (b) adverse reactions.

The index involving the largest number of qualified studies was used to assess publication bias. For common pneumonia, 30 studies that reported curative effects were used to assess publication bias, and neither Begg's test nor Egger's test found any visible evidence for publication bias (*P*_Begg_ = 0.153, *P*_Egger_ = 0.577). Since there were limited studies on curative effects for COVID-19 pneumonia, there was no assessment of publication bias for COVID-19 pneumonia. Concerning adverse reactions, neither Begg's test nor Egger's test found any visible evidence for publication bias (*P*_Begg_ = 0.904, *P*_Egger_ = 0.512). Symmetrical funnel plots are shown in Figure 4.

## 4. Discussion

Antibiotics are a traditional choice used to treat treating pneumonia; however, the inappropriate use of antibiotics leads to increasing problems such as antimicrobial resistance and is related to multiple adverse effects [61, 62]. According to recent research, the death toll will reach 10 million by 2050, due to infections caused by resistant bacteria [63]. A simple infection may be fatal in the future, and it is of crucial importance to identify and find an effective and safe alternative for treating pneumonia. Traditional Chinese medicine has been extensively used, as it offers unique advantages in regulating homeostasis and improving immunity, prevention, and control. Lianhua Qingwen is a traditional Chinese medicine that can relieve fever, act as an antitussive expectorant, dispel a cold, eliminate inflammation, provide endogenous heat, act as an antiviral agent, and improve immunity [64–66]. Some studies have reported that Lianhua Qingwen is effective against viral or bacterial infections in pneumonia [40, 46, 48, 67]; however, its principle of treatment remains inconclusive. Animal experiments have shown that Lianhua Qingwen plays a protective role in lung tissue damage by inhibiting inflammatory cell infiltration and improving protein expression in alveolar epithelial and pulmonary vascular endothelial cells [68]. Pharmacological experiments have indicated that a high dosage of Lianhua Qingwen alters the effectiveness of cyclooxygenase-2 in the arachidonic acid metabolic pathway and decreases decrease virus replication, producing significant improvements in lung inflammation [69]. It was recently reported that Lianhua Qingwen plays a significant role in the treatment of pneumonia by significantly reducing pathological changes, which might be correlated with the levels of enzymes regulated by Lianhua Qingwen, such as glutathione peroxidase, lactate dehydrogenase, superoxide dismutase, and malonaldehyde [65]. Previous studies reviewed historical records and prevention measures against SARS, H1N1 influenza, and Middle East respiratory syndrome (MERS) and reported that traditional Chinese medicine might be an alternative for the prevention and treatment of COVID-19 pneumonia [70–73]. Lu et al. [74] reported that Lianhua Qingwen plays an active role in controlling COVID-19 pneumonia, especially mild symptoms.

Our meta-analysis was performed to compare the effect of Lianhua Qingwen and conventional drugs on pneumonia symptoms. For common pneumonia, this study observed that the Lianhua Qingwen group was associated with shorter durations of flu-like symptoms, sputum, pulmonary rale, pulmonary imaging improvement, and healing period than conventional drugs; a statistically significant curative effect was also identified. The subgroup analysis based on flu-like symptoms showed statistically significant differences in fever and cough; sensitivity analysis showed that the pooled results related to common pneumonia were stable. A meta-analysis by Zhou et al. [75] revealed that Lianhua Qingwen combined with another treatment for patients with community-acquired pneumonia could improve the effective rate and pulmonary imaging and shorten durations of fever and cough, consistent with our meta-analysis. However, our meta-analysis was more comprehensive, as more new studies were included, and other important indicators of pneumonia, such as pulmonary rale, sputum, and healing period, were also taken into account. For COVID-19 pneumonia, we found that Lianhua Qingwen was more useful for improving flu-like symptoms, sputum, shortness of breath, chest tightness, pulmonary imaging improvement, curative effect, healing period, and conversion of severe cases than conventional drugs. Subgroup analysis indicated statistically significant improvements in fever, cough, fatigue, and muscle pain; however, sensitivity analysis revealed unstable results related to sputum, chest tightness, and pulmonary imaging improvement. This inconsistency might be due to the limited number of eligible studies. Only three qualified studies that reported curative effects of typical clinical symptoms were identified, and a further stratified analysis was unavailable for the meta-analysis. Several recent studies support our conclusion that Lianhua Qingwen has a positive effect on the recovery of common pneumonia and COVID-19 pneumonia. Runfeng et al. [67] discovered changes in cytokine profiles, suggesting that Lianhua Qingwen exerts an inhibitory effect on cytokine storms induced by COVID-19 pneumonia and demonstrating that Lianhua Qingwen acts as an anti-coronavirus agent by reducing cytokine release from host cells and inhibiting virus replication. Liu et al. [76] researched the efficacy and safety of integrated Chinese and Western medicines in the treatment of COVID-19 pneumonia and showed that Lianhua Qingwen improved the total effective rate (RR = 1.26, 95% CI = 1.01 to 1.56, *P* = 0.037), fever disappearance rate (RR = 1.41 95% CI = 1.21 to 1.78, *P* = 0.003), fatigue disappearance rate (RR = 1.69, 95% CI = 1.05 to 2.72, *P* = 0.032), muscle pain disappearance rate (RR = 2.91, 95% CI = 1.14 to 7.38, *P* = 0.025), and sputum disappearance rate (RR = 4.17, 95% CI = 1.59 to 10.89, *P* = 0.004). However, no other analysis was performed to detect possible sources of heterogeneity and assess the stability of the results. Even though the efficacy and outcome indicators were not as complete as ours, the results support our results to a certain extent.

Our study found a statistically significant difference in adverse reactions between the Lianhua Qingwen group and the conventional drug group. Although sensitivity analysis revealed changes in adverse reactions, subgroup analysis showed no significant difference concerning common pneumonia. Yiling Pharmaceutical, which is responsible for research on the production of Lianhua Qingwen, has reported no genotoxicity or nephrotoxicity of Lianhua Qingwen according to systemic toxicology studies [77]. A meta-analysis focused solely on the safety of Lianhua Qingwen that involved 40 RCTs reported that the incidence of adverse reactions in the Lianhua Qingwen group was significantly lower than that in the control group (RR = 0.562, 95% CI = 0.412–0.767, *P* value not reported). Subgroup analysis showed that the incidence of digestive system adverse reactions in the Lianhua Qingwen group was also lower than that in the control group (RR = 0.70, 95% CI = 0.50 to 0.97, *P* value not reported), suggesting the safety of Lianhua Qingwen [78]. Previous studies have also shown that Lianhua Qingwen has therapeutic effects on influenza. Three meta-analyses explored the efficacy/safety of Lianhua Qingwen and conventional drugs for treating influenza. Among them, Wang et al. [79] showed that Lianhua Qingwen has a superior curative effect on the treatment of viral influenza, but the incidence of adverse reactions was higher than that of conventional drugs; therefore, clinicians should pay should pay close attention to the safety of Lianhua Qingwen. Niu et al. [80] reported that Lianhua Qingwen was highly safe and had a better curative effect on the treatment of influenza than oseltamivir, ribavirin, ammonia yellow capsules, and even Huaqing Pesti capsules. Zhao et al. [81] concluded that Lianhua Qingwen was better than oseltamivir in improving the symptoms of influenza A virus infection, and no significant drug-related adverse reactions were observed in any of the included studies. Thus, these findings suggest that Lianhua Qingwen exerts better therapeutic effects by improving disease symptoms than conventional drugs, but the safety of Lianhua Qingwen cannot be ignored. Additional studies with a focus on the safety and association of Lianhua Qingwen with diseases should be performed.

Several limitations to our meta-analysis should be noted. First, the quality of the included studies was generally moderate, and only seven studies on COVID-19 pneumonia were included; thus, more high-quality research is needed. Second, the results of some indicators of COVID-19 pneumonia and adverse reactions were unstable. Since the number of studies on COVID-19 pneumonia were limited, publication bias and meta-regression analysis have not been performed. Finally, although English and Chinese articles were retrieved, only Chinese studies were qualified; therefore, language bias might not be excluded.

Some advantages should be mentioned. First, to our knowledge, this is the most comprehensive meta-analysis of the efficacy of Lianhua Qingwen for the treatment of common pneumonia and COVID-19 pneumonia. Second, to our knowledge, this is the first meta-analysis of the use of Lianhua Qingwen for the treatment of pneumonia based on several indicators (i.e., sputum, curative effect, and healing period for common pneumonia; flu-like symptoms, shortness of breath, breathlessness, chest tightness, pulmonary imaging improvement, healing period, and conversion of severe cases for COVID-19 pneumonia). Finally, we determined that Lianhua Qingwen combined with conventional drugs can better promote the recovery of certain clinical symptoms related to common pneumonia and COVID-19 pneumonia than conventional drugs.

## 5. Conclusion

Lianhua Qingwen combined with conventional drugs may be a promising therapy for treating pneumonia, including common pneumonia and COVID-19 pneumonia, but more high-quality studies are needed to explore the efficacy and mechanism of Lianhua Qingwen in treating pneumonia.

## Figures and Tables

**Table 1:** Main characteristics and quality score of the included studies for common pneumonia and COVID-19 pneumonia.

Type	Study	Year	Group	Age	Sex		Efficacy evaluation	Adverse reactions	Quality score
				T/C	T (M/F)	C (M/F)			
Common pneumonia	Chen	2020	R	5.45 ± 0.55/5.37 ± 0.38	52 (26/26)	52 (23/29)	Curative effect, fever, cough, pulmonary rale	Gastrointestinal reaction, back pain, cry and scream, headache	4
	Chen	2020	I	7.81 ± 1.64/7.77 ± 1.53	79 (45/37)	78 (42/36)	Curative effect, fever, cough	Diarrhoea, tachycardia, facial redness, headache, rash	2
	Feng	2020	R	44.5 ± 10.7/43.6 ± 10.1	37 (19/18)	37 (20/17)	Curative effect, healing period	Not reported	4
	Wang	2019	R	5.55 ± 1.68/5.28 ± 1.53	80 (44/36)	80 (42/38)	Curative effect	Not reported	4
	Lin	2019	R	7.33 ± 2.42/4.34 ± 1.41	53 (30/23)	50 (28/25)	Curative effect, fever, cough, sputum, pulmonary rale, healing period	Not reported	4
	Sun	2019	I	51.06 ± 5.92/50.17 ± 5.68	41 (28/13)	41 (27/14)	Curative effect, cough, fever, pulmonary rale	Nausea, vomiting, diarrhoea	2
	Xia	2019	H	19.5 ± 2.1	55	55	Curative effect, cough, fever, Healing period, pulmonary rale	Vomiting, rash, dizziness	2
	Hu	2018	R	43.4 ± 4.8/44.7 ± 4.1	52 (37/15)	52 (36/16)	Curative effect, cough, fever, pulmonary rale, healing period	Gastrointestinal reaction, loss of appetite, vomiting	4
	Lv	2018	R	48.42 ± 7.59/48.61 ± 7.73	60 (29/31)	60 (27/33)	Curative effect, cough, fever, pulmonary rale	Stomach ache, nausea, rash	4
	Chen	2018	R	6.44 ± 3.50	59	58	Fever, cough, pulmonary rale, healing period	No adverse reaction	4
	Zhang	2018	R	69.4 ± 2.1/69.2 ± 2.0	39 (24/15)	39 (23/16)	Curative effect, cough, sputum, pulmonary rale	Increased gamma glutamyl aminotransferase, hypotension, edema	3
	Zhang	2018	R	46.81 ± 9.22/46.91 ± 9.34	60 (32/28)	60 (33/27)	Curative effect, fever, cough, pulmonary rale	Diarrhoea, rash, elevated aspartate transferase	3
	Liu	2017	R	40.34 ± 9.23/42.13 ± 8.18	53 (30/23)	53 (32/21)	Curative effect, fever, cough, pulmonary rale	No adverse reaction	3
	Han	2017	I	5.24 ± 1.03/6.27 ± 0.98	62 (31/31)	62 (33/29)	Pulmonary rale, cough, fever, healing period	Not reported	2
	Yang	2016	R	29.20 ± 5.10	38	22	Curative effect, fever, cough, pulmonary imaging improvement, healing period	Gastrointestinal reaction	3
	Wang	2015	R	61–75/59–74	34	34	Curative effect, fever, cough, pulmonary rale	Loss of appetite, skin itch, dizziness	3
	Tu	2015	R	5.3 ± 1.1/5.1 ± 1.3	50 (30/20)	50 (28/22)	Fever, cough, pulmonary rale, healing period	Not reported	3
	Xu	2015	R	35.6 ± 12.8/34.8 ± 13.2	38 (18/20)	30 (14/16)	Fever, healing period	No specific type	3
	Meng	2015	R	50.7 ± 3.4/51.4 ± 4.2	20 (12/8)	18 (10/8)	Curative effect, fever, cough	Nausea, rash	4
	Zhu	2015	R	47.0 ± 3.0/48.5 ± 3.5	31 (23/8)	31 (21/10)	Curative effect, fever, cough, sputum, pulmonary imaging improvement	Rash, leukopenia, gastrointestinal reaction	4
	Li	2014	R	45.3 ± 1.3/44.6 ± 1.2	43 (27/16)	43 (25/18	Curative effect, cough, fever, pulmonary imaging improvement, healing period	No adverse reaction	3
	Li	2014	R	45.4/44.3	25 (13/12)	24 (12/12)	Curative effect	No adverse reaction	3
	Jiang	2014	R	18–65	60 (32/28)	60 (38/22)	Curative effect, fever, cough, pulmonary rale, pulmonary imaging improvement	Diarrhoea	3
	Liu	2014	R	61.82 ± 18.50/59.23 ± 13.50	52 (30/22)	52 (32/20)	Curative effect, fever, cough, pulmonary rale, pulmonary imaging improvement	No adverse reaction	3
	Gao	2014	R	48.2 ± 6.7/51.3 ± 4.8	60 (34/26)	60 (33/27)	Curative effect, fever, cough, pulmonary imaging improvement, healing period	Rash, white blood cell index returned to normal, gastrointestinal reaction	3
	Zou	2014	R	76.5 ± 5.2/77.2 ± 5.9	30 (18/12)	30 (20/10)	Curative effect, fever, cough, pulmonary rale, sputum	Nausea, vomiting	3
	Dong	2014	R	71.64 ± 11.73/72.58 ± 10.69	40 (21/19)	42 (24/18)	Curative effect, fever, cough, sputum	No adverse reaction	3
	Wang	2014	R	36.3 ± 7.50/35.6 ± 7.40	40 (26/14)	38 (24/14)	Curative effect, fever	No adverse reaction	3
	Wang	2014	R	3 to 15	30 (19/11)	30 (17/13)	Curative effect, fever, cough, pulmonary rale	No adverse reaction	3
	Zhou	2014	R	35.3 ± 7.60/30.10 ± 7.50	46 (26/20)	41 (19/22)	Curative effect, fever	No adverse reaction	3
	Wang	2013	R	18 to 65	50	50	Curative effect, fever, cough, pulmonary imaging improvement, healing period	Not reported	3
	Chen	2013	R	71 ± 8.6	76 (44/32)	76 (42/34)	Curative effect, fever, cough, pulmonary rale, healing period	Nausea, diarrhoea, rash	3
	Peng	2013	R	33.6 ± 11.2/32.8 ± 10.1	30 (16/14)	30 (15/15)	Curative effect	No adverse reaction	5
	Zhong	2013	R	35.3 ± 7.60/30.10 ± 7.50	38 (20/18)	32 (17/15)	Curative effect	No adverse reaction	3
	Xu	2012	R	28.30 ± 6.80/29.20 ± 6.50	32 (18/14)	30 (16/14)	Curative effect, fever	No adverse reaction	3
COVID-19 pneumonia	Cheng	2020	I	55.5 ± 12.3/55.8 ± 11.6	51 (26/25)	51 (27/24)	Fever, cough, fatigue, muscle pain, Sputum, shortness of breath, chest tightness, pulmonary imaging improvement, breathlessness, nausea, loss of appetite	Not reported	2
	Hu	2020	R	50.4 ± 15.2/51.8 ± 14.8	142 (79/63)	142 (71/71)	Curative effect, pulmonary imaging improvement, conversion of severe cases	Abnormal liver function, renal insufficiency, headache, nausea, vomiting, diarrhoea, loss of appetite	5
	Lv	2020	I	59.1 ± 16.56/60.2 ± 17.01	63 (28/35)	38 (18/20)	Fever, cough, fatigue, muscle pain, sputum, sore throat, shortness of breath, chest tightness, breathlessness, headache, nausea, loss of appetite, diarrhoea	No adverse reaction	2
	Shi	2020	I	47.94 ± 14.46/46.72 ± 17.40	49 (26/23)	18 (10/8)	Curative effect, fever, healing period, pulmonary imaging improvement, conversion of severe cases	Not reported	2
	Xia	2020	I	54.18 ± 13.08/53.67 ± 12.70	34 (17/17)	18 (6/12)	Curative effect, healing period, pulmonary imaging improvement, conversion of severe cases	No adverse reaction	2
	Yao	2020	I	57.1 ± 14.0/62.4 ± 12.3	21 (16/5)	21 (12/9)	Fever, cough, fatigue, muscle pain, sputum, sore throat, shortness of breath, chest tightness, breathlessness, headache, nausea, loss of appetite, diarrhoea	Not reported	2
	Yu	2020	R	48.27 ± 9.56/47.25 ± 8.67	147 (82/65)	147 (89/59)	Curative effect, pulmonary imaging improvement, conversion of severe cases	No adverse reaction	4

R, randomization; I, intervention; H, hospitalization order; S, subject; T, treatment group; C, control group; M, male; F, female.

**Table 2:** The quantitative analysis of common pneumonia and COVID-19 pneumonia.

Subject	Efficacy index	Effect value	95% CI	*P*_Effect value_	Heterogeneity		M
					*I* ^2^	*P*	
Common pneumonia	**Flu-like symptoms**	WMD = −1.81	−2.12, −1.50	<0.001	98.0	<0.001	R
	Fever	WMD = −1.58	−1.99, −1.17	<0.001	98.3	<0.001	R
	Cough	WMD = −2.07	−2.57, −1.57	<0.001	97.7	<0.001	R
	**Sputum**	WMD = −1.10	−1.50, −0.70	<0.001	62.2	0.047	R
	**Pulmonary rales**	WMD = −2.03	−2.74, −1.31	<0.001	97.1	<0.001	R
	Pulmonary imaging improvement	WMD = −1.88	−2.28, −1.47	<0.001	56.5	0.032	R
	Curative effect	OR = 3.65	2.81, 4.76	<0.001	0.0	0.945	F
	Healing period	WMD = −1.68	−2.62, −0.74	<0.001	97.0	<0.001	R

COVID-19 pneumonia	Flu-like symptoms	OR = 3.18	2.36, 4.29	<0.001	6.7	0.367	F
	Fever	OR = 4.05	1.78, 6.59	<0.001	0.0	0.548	F
	Cough	OR = 3.43	1.87, 6.29	<0.001	7.7	0.338	F
	Sore throat	OR = 0.31	0.03, 2.99	0.314	39.8	0.197	F
	Nausea	OR = 1.98	0.49, 7.95	0.336	7.4	0.340	F
	Diarrhoea	OR = 1.28	0.18, 9.30	0.810	0.0	0.638	F
	Loss of appetite	OR = 6.05	0.69, 53.14	0.104	74.5	0.020	R
	Fatigue	OR = 2.82	1.44, 5.53	0.003	0.0	0.757	F
	Muscle pain	OR = 5.01	1.44, 17.40	0.011	0.0	0.723	F
	Headache	OR = 2.67	0.30, 23.83	0.378	0.0	0.938	F
	Sputum	OR = 4.30	1.01, 18.22	0.048	61.9	0.073	R
	**Shortness of breath**	OR = 10.62	3.71, 30.40	<0.001	0.0	0.751	F
	**Breathlessness**	OR = 4.81	0.97, 23.86	0.055	0.0	0.672	F
	**Chest tightness**	OR = 3.02	1.23, 7.42	0.016	31.5	0.232	F
	**Pulmonary imaging improvement**	OR = 1.77	1.29, 2.42	<0.001	43.7	0.130	F
	**Curative effect**	OR = 2.49	1.76–3.53	<0.001	29.8	0.234	F
	**Healing period**	WMD = −2.06	−3.36, 0.75	0.002	0.0	0.810	F
	**Conversion of severe cases**	OR = 0.46	0.27, 0.77	0.003	18.2	0.295	F

All	**Adverse reactions**	OR = 0.74	0.56, 0.97	0.029	0.0	0.994	F
	Common pneumonia	OR = 0.75	0.54, 1.05	0.097	0.0	0.992	F

M, model; R, random-effects model; F, fixed-effects model.

**Table 3:** Sensitivity analysis of common pneumonia and COVID-19 pneumonia.

Subject	Efficacy index	Effect value	95% CI
Common pneumonia	Flu-like symptoms	WMD = −1.81	−2.17, −1.47
	Sputum	WMD = −1.10	−1.94, −0.52
	Pulmonary rales	WMD = −2.03	−2.87, −1.21
	Pulmonary imaging improvement	WMD = −1.88	−2.45, −1.37
	Curative effect	OR = 3.65	2.69, 5.13
	Healing period	WMD-1.68	−2.80, −0.53
	Adverse reaction	OR = 0.75	0.50, 1.19

COVID1-19 pneumonia	Flu-like symptoms	OR = 3.18	2.18, 4.63
	Sputum^*∗*^	OR = 4.30	0.36, 47.40
	Shortness of breath	OR = 10.62	2.80, 66.79
	Breathlessness	OR = 4.81	0.29.42.01
	Chest tightness^*∗*^	OR = 3.02	0.77, 28.11
	Pulmonary imaging improvement^*∗*^	OR = 1.77	0.95, 3.41
	Curative effect	OR = 2.49	1.58, 4.85
	Healing period	WMD = −2.06	−4.01, 0.01
	Conversion of severe cases	OR = 0.46	0.10, 0.92

All	Adverse reactions^*∗*^	OR = 0.74	0.54, 1.05

^*∗*^The sensitivity analysis results indicated that deleting individual studies would change the combined results of the meta-analysis.

**Table 4:** Results of the meta-regression.

Subject		Publication year	Sample size	Quality score
Common pneumonia	Flu-like symptoms	*P* = 0.190	*P* = 0.070	*P* = 0.330
	Sputum	*P* = 0.947	*P* = 0.823	*P* = 0.131
	Pulmonary rales	*P* = 0.150	*P* = 0.480	*P* = 0.510
	Pulmonary imaging improvement	*P* = 1.000	*P* = 0.770	*P* = 0.960
	Healing period	*P* = 0.850	*P* = 0.990	*P* = 0.150

## Data Availability

The data used to support the findings of this study are available from the corresponding author upon request.

## Supplementary Materials

Supplementary Materials Figure S1: forest plot of the comparison between the Lianhua Qingwen group and the conventional drug group on other symptoms related to common pneumonia. Figure S2: forest plot of the comparison between the Lianhua Qingwen group and the conventional drug group on other symptoms related to COVID-19 pneumonia.
