# Supplementary material for: Efficacy of Lianhua Qingwen Compared with Conventional Drugs in the Treatment of Common Pneumonia and COVID-19 Pneumonia: A Meta-Analysis
Source: Evid Based Complement Alternat Med. 2020 Sep 17;2020:5157089. doi: 10.1155/2020/5157089 (PMC7501551; doi:10.1155/2020/5157089)
Supplement: Supplementary Materials — Figure S1: forest plot of the comparison between the Lianhua Qingwen group and the conventional drug group on other symptoms related to common pneumonia. Figure S2: forest plot of the comparison between the Lianhua Qingwen group and the conventional drug group on other symptoms related to COVID-19 pneumonia. [file 5157089.f1.zip › 5157089.f1/Figure S1.docx]

Figure S1. Forest plot of the comparison between the Lianhua Qingwen group and the conventional drug group on other symptoms related to common pneumonia.

a, sputum; b, pulmonary rales; c, pulmonary imaging improvement; d, curative effect; e, healing period.
